# Stressful Life Events and Resilience During the COVID-19 Lockdown Measures in Italy: Association With Mental Health Outcomes and Age

**DOI:** 10.3389/fpsyt.2021.635832

**Published:** 2021-03-08

**Authors:** Rodolfo Rossi, Tommaso B. Jannini, Valentina Socci, Francesca Pacitti, Giorgio Di Lorenzo

**Affiliations:** ^1^Department of Systems Medicine, University of Rome Tor Vergata, Rome, Italy; ^2^Department of Biotechnological and Applied Clinical Sciences, University of L'Aquila, L'Aquila, Italy; ^3^Istituto di Ricovero e Cura a Carattere Scientifico (IRCCS) Fondazione Santa Lucia, Rome, Italy

**Keywords:** old age, resilience, COVID-19, mental health, stress

## Abstract

**Background:** The COVID-19 pandemic, due to its disproportionated higher morbidity and mortality rates in the older age, has been considered to be a “geropandemic.” Several studies, however, have found that older age is associated with lower psychological distress in relation to the COVID-19 outbreak and related lockdown measures.

**Aim:** To explore the role of Resilience as a mediator between stressful COVID-19 related life events and depressive and, anxiety symptoms and perceived stress, and to ascertain the role of age as a moderator of the mediator's effect.

**Methods:** An on-line survey was spread through social networks during the first lockdown in Italy. Depressive and anxiety symptoms and perceived stress were measured using the Italian version of the Patient Health Questionnaire-9 (PHQ-9), the Generalized Anxiety Disorder Questionnaire-7 (GAD-7) and the Perceived Stress Scale (PSS). Resilience was measured using the Italian version of the Resilience Scale for Adults (RSA). Stressful COVID-19 related life events were explored using a checklist of events derived from the International Adjustment Disorder Questionnaire (IADQ). After a preliminary panel of linear regressions, mediation was tested using Structural Equation Modeling and inspecting the bootstrapped indirect effects. Afterwards, age was introduced as a mediator of the indirect effect in a moderated mediation analysis.

**Results:** Twenty one thousand three hundred and thirty four subjects completed the questionnaire, 17,178 (80.52%) were female, 748 (3.5%) were >60 years old. In the whole sample, the presence of any stressful event was associated with depressive and anxiety symptoms and perceived stress. Resilience mediated the effects of stressful COVID-19-related events on depressive and anxiety symptoms and perceived stress. The moderated mediation analysis revealed that age moderated the mediation effect of Resilience between the presence of a stressful event and the selected outcomes.

**Conclusion:** Taken together, our results show that age moderates the mediating effect of Resilience in the relationship between COVID-19-related stressful events and depressive and anxiety symptoms and perceived stress. Older adults' Resilience was less influenced by stressful events, and this could be one of the reasons accounting for the better mental health outcomes observed in the older age.

## Introduction

The COVID-19 pandemic, due to its disproportionated higher morbidity and mortality rates in the older age, has been considered to be a “geropandemic” (1). Several studies, however, have found that older age is associated with lower psychological distress in relation to the COVID-19 outbreak and lockdown measures. Younger individuals, especially women, report higher levels of depressive, anxious and stress-related symptoms compared to older age (2–4). The evidence that older age is associated with better psychological outcomes is in apparent contrast with the increased physical vulnerability of the elderly to the COVID-19. Despite morbidity and mortality is highly correlated with age (5), a recent study found that older adults show slightly less COVID-19-related worries compared to younger participants (2). On the other hand, several studies have highlighted issues such as isolation and loneliness.

Resilience could be one of the putative psychological factors that could account for a better adaptation to the COVID-19 pandemic in the elderly. In the geriatric literature, resilience is associated with successful aging (6), lower mortality, lower depressive symptoms, increased quality of life and better lifestyle behaviors.

Resilience is considered a protective mechanism operating in the face of negative stressors (7), and it is constantly associated with better psychological well-being and lower mental illness. It has been suggested that older adults may express higher levels of resilience compared to the younger ones (8), in particular regarding emotion regulation and problem-solving dimensions. In contrast, younger individuals show slightly higher social support, in the context of reduced overall resilience levels. According to a recent systematic review, resilience in older adults could be operationalized as a 4-dimension construct, that includes intrapersonal, interpersonal, spiritual and experiential protective factors (9). It is noteworthy that these factors are differentially associated with age or environmental circumstances, being interpersonal factors the most volatile over time and spiritual and experiential factors being associated with older age.

Age differences in resilience levels could be associated with the difference in mental health outcomes across the general population. It has been reported that during the lockdown, resilience levels were lower than normative data in younger adults aged 18–35 years (10), and this was suggested as one key factor affecting the general population's mental health and pessimism about the future of the pandemic.

Few studies have addressed the relation between resilience, mental health and age at the time of the COVID-19 pandemic. In a recent study on a US sample, higher resilience was associated with lower depressive and anxiety symptoms in the general population (2). In his study, the mitigating effect of high resilience with lower anxiety was stronger in older age. Such finding was associated with lower COVID-19-related worries in the older age. In another study on a Turkish sample, older age was associated with both higher resilience and lower depression rates (11).

The exact pathways by which resilience interacts with age in affecting mental health remains unclear. The present study aims to address the role of resilience in older adults with respect to mental health outcomes during the COVID-19 pandemic. Our hypothesis is that the role of Resilience in mitigating the impact of COVID-related stressful life events may be different at different ages. To test this hypothesis, it was firstly tested whether resilience would mediate the relationship between COVID-19-related stressful events and depressive and anxiety symptoms and perceived stress (mediation). Secondly, we tested whether the indirect effect of stressful events on depressive and anxiety symptoms and perceived stress via resilience would be moderated by age. Such model is referred to as Moderated Mediation, i.e., a model in which a mediator has a different effect at different levels of a moderating variable (12).

## Methods

### Study Design

This study is a cross-sectional web-based observational study, and it is a part of a long-term monitoring program of mental health outcomes in the general population and health care workers. On-line consent was obtained from the participants. At 3 weeks after the beginning of the lockdown, an anonymous survey was conducted among a self-selected sample from the Italian population. Every person living in Italy ≥ 18 years old was eligible. Approval for this study was obtained from IRB at the University of L'Aquila. This study adheres to the Declaration of Helsinki.

### Sampling Strategy and On-Line Questionnaire Dissemination

For the purpose of this study, the questionnaire was spread using sponsored adverts on Facebook®, as well as using a snowball spreading technique starting from the researchers' acquaintances. Because of the particular dissemination technique, it was not possible to have precise data on response rate, however using the Facebook Ads app, it was possible to estimate that the number of link clicks was about 100.000, while the ad reached nearly 1 million people.

### Outcome Measures

**The Italian version of the 9-item Patient Health Questionnaire (PHQ-9)** was used to assess depressive symptoms. PHQ-9 comprises nine depressive symptoms, rated on a 4-point Likert scale, range 0–27. The total score has been taken into consideration as a continuous variable. PHQ-9 is a widely used instrument in epidemiological research as a depression screener. In our sample, internal consistency was *a* = 0.87.

**The Italian version of the 7-item Generalized anxiety disorder questionnaire (GAD-7)** was used to assess anxiety symptoms. GAD-7 includes seven symptoms, rated on a 4-point Likert scale, range 0–21 (13). The total score has been taken into consideration as a continuous variable. GAD-7 is a widely used instrument in epidemiological research as anxiety screener. In our sample, internal consistency was *a* = 0.91.

**The Italian version of the 10-item Perceived Stress Scale (PSS)** was used to assess perceived stress. PSS includes ten items rated on a 0–4 Likert scale. In our sample, internal consistency was *a* = 0.87.

### Independent Variables, Covariates, and Confounders

**Age** was used both as a continuous and binary variable, with a cut-off of 60 years old as a separation between older and younger adults.

**Stressful events** were assessed using the International Adjustment Disorder Questionnaire (IADQ) checklist of stressful events (14). The IADQ checklist explores eight different stressful events, namely economic, job and study difficulties, problems related to housing, relational problems, own's and a loved one's health problems, caregiving problems. In the original version, each item has a yes/no response. We modified the response as follows: “*no/yes/yes, due to COVID-19 pandemic or lockdown”* in order to capture COVID-19 related stressful events. For this study, a binary variable was created with 0 = “no stressful events due to COVID-19” and 1 = “one or more stressful events due to COVID-19.”

**Resilience** was assessed using the 11-items Resilience Scale for Adults (RSA). The RSA-11 was obtained from the original 33 item version (15). Participants answer on a 7-point semantic differential scale in which each item has a positive and a negative attribute at each end of the scale continuum. For this study, the RSA-11 total score was taken into account, with higher scores indicating lower levels of Resilience.

The following potential confounders were selected: Gender; Geographical Area (Northern Italy: Aosta Valley, Piedmont, Lombardy, Liguria, Trentino-Alto Adige, Veneto, Friuli-Venezia Giulia, Emilia-Romagna; Center Italy: Tuscany, Umbria, Marche, Lazio; Southern Italy: Abruzzo, Molise, Puglia, Campania, Calabria, Basilicata, Sicily and Sardinia); Education level (lower education, undergraduate, graduate, post-graduate degree).

### Statistical Analysis

All statistical analyses were conducted using STATA® 16 (StataCorp).

Firstly, the following associations were tested using a panel of linear or logistic regressions, as appropriate for the dependent variable:

1. association between age and PHQ-9, GAD-7 and PSS
2. association between RSA and stressful events and PHQ-9, GAD-7 and PSS
3. association between RSA and stressful events and age.

Secondly, a mediation model was fitted on PHQ-9, GAD-7 and PSS, with stressful events as independent variable and RSA as mediator. Mediation was tested by bootstrapping the indirect effect at 5,000 replications. The significance of the bootstrapped indirect effects was ascertained inspecting the normal-based and bias-corrected confidence intervals.

**Figure 1:**
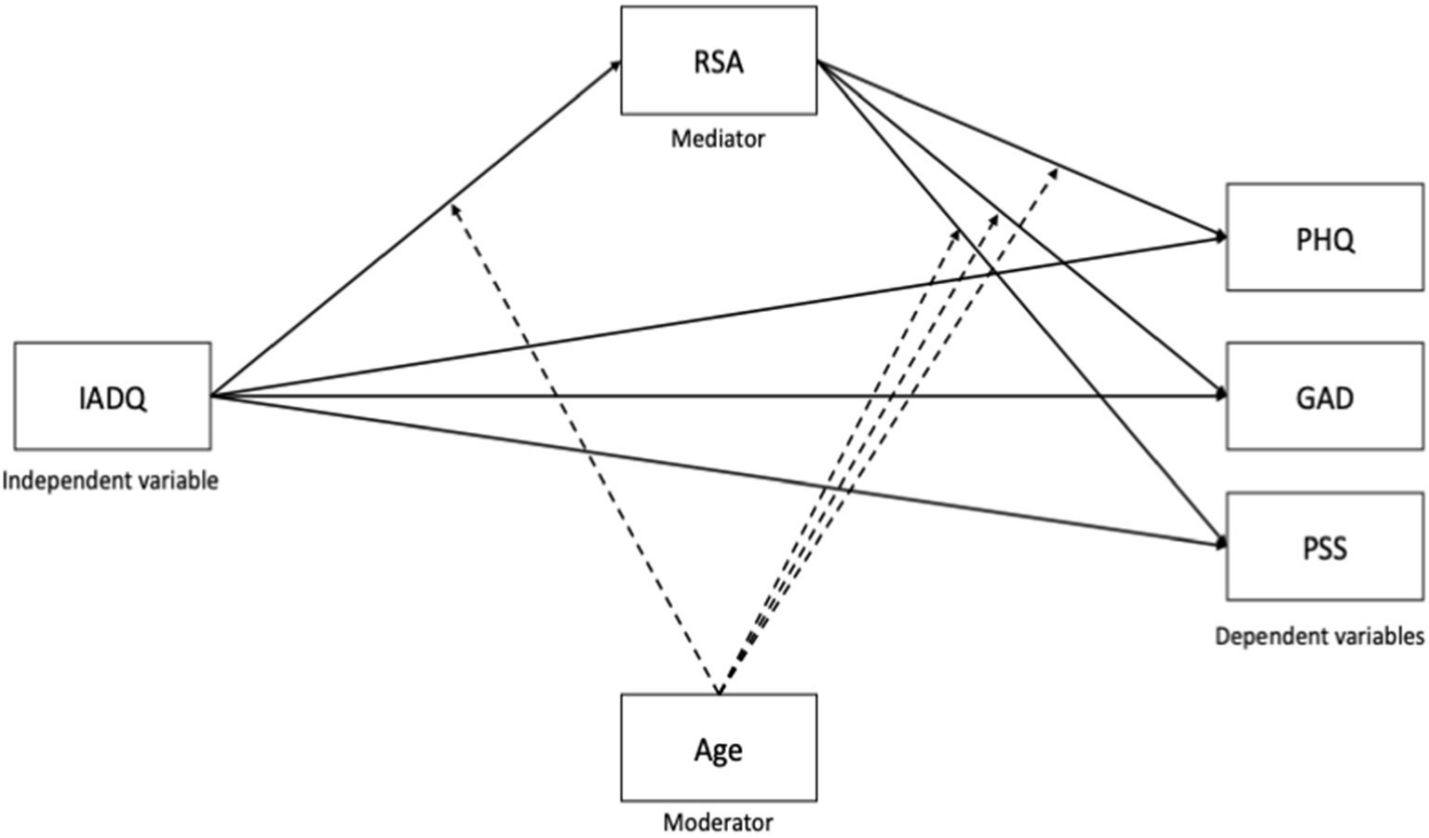
Proposed moderated mediation model. PHQ, Patient Health Questionnaire; GAD, Generalized Anxiety Questionnaire; PSS, Perceived Stress Questionnaire; RSA, Resilience Scale for Adults; IADQ, International Adjustment Disorder Questionnaire – stressful events checklist.

Finally, conditional indirect effects of COVID-19 stressful events on PHQ-9, GAD-7 and PSS via RSA, entering age as a moderator were tested. This model is referred to as “Moderated Mediation” and it is largely founded on Model 59 by Hayes (12). In Figure 1 we show the proposed model for the moderated mediation. The significance of the bootstrapped conditional indirect effects was ascertained inspecting the normal-based and bias-corrected confidence intervals.

## Results

### Sample Characteristics

Sample characteristics are reported in Table 1. Twenty-one thousand three hundred thirty-four subjects participated in the study. Of these, 17,178 (80.52%) were female. Mean age was 38.95 (SD = 12.77); 748 (3.51%) subjects were 60 or older.

**Table 1:** Sample characteristics.

	**Total sample**	** <60 yr**	**≥60 yr**	**Statistics (**χ**^2^ or Mann-Whitney as appropriate)**
	**Mean (SD)/*****N*** **(%)**	**Mean (SD)/*****N*** **(%)**	**Mean (SD)/*****N*** **(%)**	
Age	38.95 (12.77)	38.07 (12.09)	63.12 (4.62)	
Gender				$\chi_{\left(1\right)}^{2}$ = 05.66; *p =* 0.017
*Male*	4,155 (19.48%)	3,984 (19.35%)	171 (22.86%)	
*Female*	17,178 (80.52%)	16,601 (80.65%)	577 (77.14%)	
History of psychiatric disorders	6,067 (28.51%)	5,860 (28.54%)	207 (27.71%)	$\chi_{\left(1\right)}^{2}$ = 0241; *p =* 0.623
N° of Stressful events	0.81 (1.14)	0.83 (1.14)	0.35 (0.77)	*z* = 12.32; *p* < 0.001
PHQ	10.67 (6.39)	10.78 (6.38)	7.79 7.79)	*z* = 12.84; *p* < 0.001
GAD	9.03 (5.95)	9.13 (5.95)	6.26 (5.36)	*z* = 13.24; *p* < 0.001
PSS	24.60 (8.40)	24.76 (8.37)	20.38 (8.06)	*z* = 13.85; *p* < 0.001
RSA	36.96 (11.84)	37.04 (11.81)	34.68 (12.32)	*z* = 5.40; *p* < 0.001

*PHQ, Patient Health Questionnaire; GAD, Generalized Anxiety Questionnaire; PSS, Perceived Stress Questionnaire; RSA, Resilience Scale for Adults*.

### Associations Between Selected Variables

Tables 2–4 report the linear associations between age, resilience, COVID-19 related stressful events and psychopathology. In our sample, age was inversely associated with PHQ-9, GAD-7 and PSS (Table 2). This association held after adjusting for the selected confounders. The presence of any stressful event was associated with PHQ-9, GAD-7 and PSS (Table 3). Better resilience resources (i.e., a lower score on the RSA-11) was inversely associated with PHQ-9, GAD-7 and PSS. These associations held after adjusting for the selected confounders.

**Table 2:** Association between age and depression, anxiety and stress.

	**Unadjusted**		**Adjusted^§^**	
	**b (95% CI)**	***p***	**b (95% CI)**	***p***
Age → PHQ Total	−0.11 (−0.12, −0.10)	<0.001	−0.12 (−0.12, −0.11)	<0.001
Age → GAD Total	−0.098 (−0.10, −0.09)	<0.001	−0.10 (−0.11, −0.10)	<0.001
Age → PSS Total	−0.16 (−0.17, −0.16)	<0.001	−0.17 (−0.18, −0.16)	<0.001

§ *adjusted for gender, region and education level. PHQ, Patient Health Questionnaire; GAD, Generalized Anxiety Questionnaire; PSS, Perceived Stress Questionnaire*.

**Table 3:** Association between risk and protective factors and depression, anxiety and stress.

	**Unadjusted**		**Adjusted^§^**	
	**b (95% CI)**	***p***	**b (95% CI)**	***p***
Any IADQ → PHQ Total	0.44 (0.41, 0.46)	<0.001	0.42 (0.39, 0.44)	<0.001
Any IADQ → GAD Total	0.42 (0.39, 0.44)	<0.001	0.40 (0.37, 0.42)	<0.001
Any IADQ → PSS Total	0.46 (0.43, 0.48)	<0.001	0.43 (0.41, 0.46)	<0.001
RSA → PHQ Total	0.58 (0.57, 0.59)	<0.001	0.57 (0.56, 0.58)	<0.001
RSA → GAD Total	0.52 (0.50, 0.53)	<0.001	0.51 (0.50, 0.52)	<0.001
RSA → PSS Total	0.54 (0.53, 0.55)	<0.001	0.54 (0.52, 0.55)	<0.001

*PHQ, Patient Health Questionnaire; GAD, Generalized Anxiety Questionnaire; PSS, Perceived Stress Questionnaire; RSA, Resilience Scale for Adults; IADQ, International Adjustment Disorder Questionnaire – stressful events checklist. RSA, PHQ, GAD, and PSS are standardized values*.

§ *adjusted for gender, region and education level*.

**Table 4:** Association between risk and protective factors and age.

	**Unadjusted**		**Adjusted^§^**	
	**b (95% CI)**	***p***	**b (95% CI)**	***p***
^*^Age → Any IADQ	−0.44 (−0.47, −0.41)	<0.001	−0.46 (−0.49, −0.43)	<0.001
Age → RSA	−0.14 (−0.16, −0.13)	<0.001	−0.15 (−0.16, −0.13)	<0.001
^*^Old Age → Any IADQ	−1.00 (−1.17, −0.83)	<0.001	−1.00 (−1.17, −0.82)	<0.001
Old Age → RSA	−0.20 (−0.27, −0.12)	<0.001	−0.21 (−0.29, −0.14)	<0.001

* *logit function, dependent variable is binary*.

§ *adjusted for gender, region and education level. RSA, Resilience Scale for Adults; IADQ, International Adjustment Disorder Questionnaire – stressful events checklist*.

Finally, age was associated with lower odds of endorsing any COVID-19-related stressful event and with better resilience (Table 4). These associations held after adjusting for the selected confounders.

### Mediation Analysis

**Figure 2:**
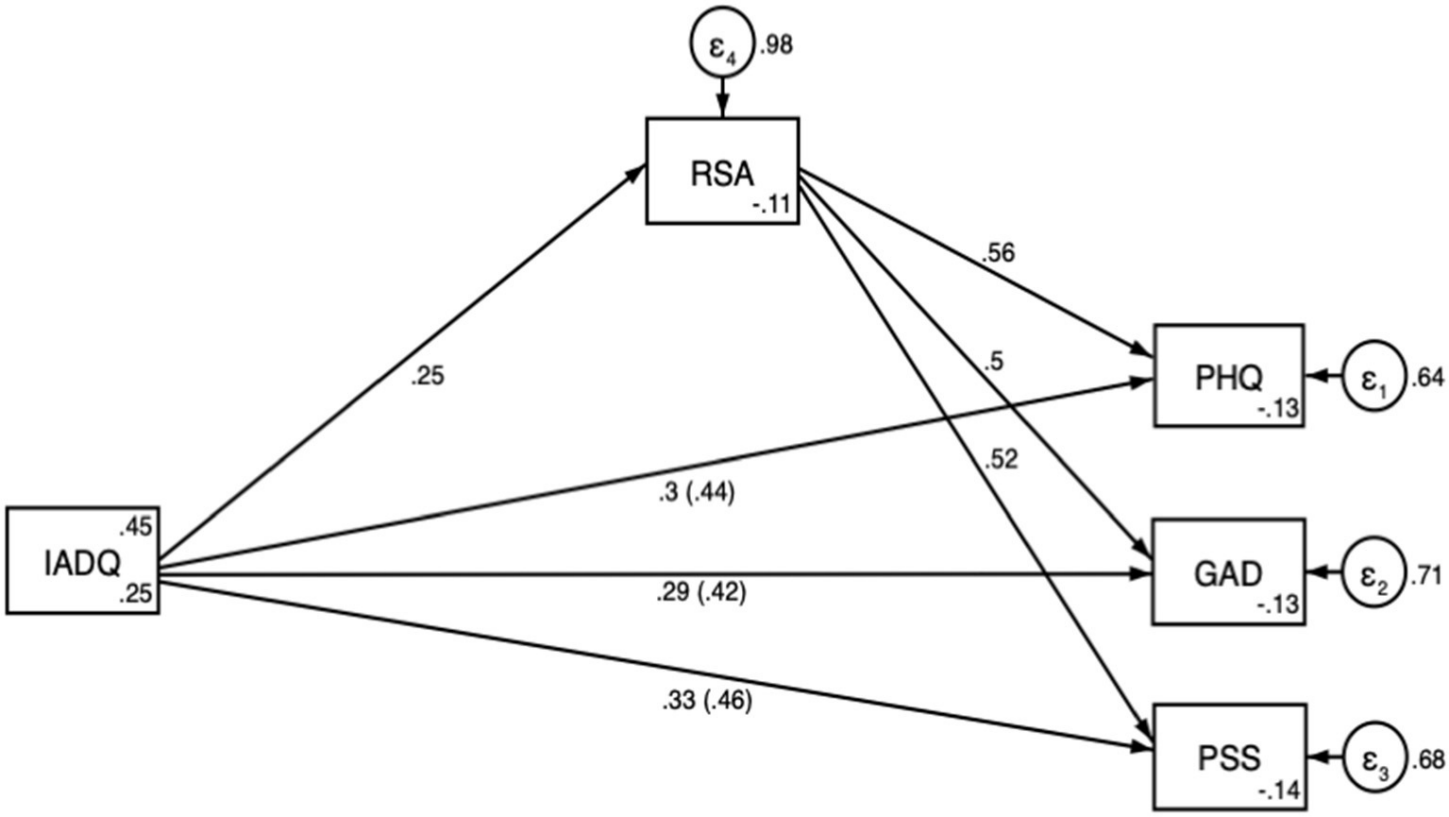
Mediation analysis path diagram with direct effects and, in parenthesis, total effects. PHQ, Patient Health Questionnaire; GAD, Generalized Anxiety Questionnaire; PSS, Perceived Stress Questionnaire; RSA, Resilience Scale for Adults; IADQ, International Adjustment Disorder Questionnaire – stressful events checklist.

Mediation analysis (Figure 2 and Table 5) showed that the impact of COVID-related stressful events on PHQ-9, GAD-7 and PSS was very similar. RSA partially mediated the impact of stressful events on the selected outcomes, as confirmed by inspection of the Bootstrapped confidence intervals of the indirect effect through RSA.

**Table 5:** Path analysis results.

	**Coef**	**Confidence intervals**
**Direct effects**		
RSA → PHQ	0.56 (0.55, 0.57)	(0.55, 0.57)
IADQ → PHQ	0.30 (0.27, 0.32)	(0.27, 0.32)
RSA → GAD	0.50 (0.49, 0.51)	(0.49, 0.51)
IADQ → GAD	0.29 (0.27, 0.31)	(0.27, 0.31)
RSA → PSS	0.52 (0.51, 0.53)	(0.51, 0.53)
IADQ → PSS	0.33 (0.30, 0.35)	(0.30, 0.35)
IADQ → RSA	0.25 (0.22, 0.28)	(0.22, 0.28)
**Total effects**
RSA → PHQ	0.56 (0.55, 0.57)	(0.55, 0.57)
IADQ → PHQ	0.42 (0.41, 0.46)	(0.41, 0.46)
RSA → GAD	0.50 (0.49, 0.51)	(0.49, 0.51)
IADQ → GAD	0.42 (0.39, 0.44)	(0.39, 0.44)
RSA → PSS	0.52 (0.51, 0.53)	(0.51, 0.53)
IADQ → PSS	0.46 (0.43, 0.48)	(0.43, 0.48)
IADQ → RSA	0.25 (0.22, 0.28)	(0.22, 0.28)
**Bootstrapped indirect effects**
IADQ → (RSA) → PHQ	0.14	0.12, 0.15 (*N*)
		0.12, 0.16 (BC)
IADQ → (RSA) → GAD	0.12	0.11, 0.14 (*N*)
		0.11, 0.14 (BC)
IADQ → (RSA) → PSS	0.13	0.11, 0.14 (*N*)
		0.11, 0.14 (BC)

*(N), Normal-based (95% Conf. Interval); (BC), Bias-corrected confidence interval; PHQ, Patient Health Questionnaire; GAD, Generalized Anxiety Questionnaire; PSS, Perceived Stress Questionnaire; RSA, Resilience Scale for Adults; IADQ, International Adjustment Disorder Questionnaire – stressful events checklist*.

### Moderated Mediation Analysis

Table 6 reports the bootstrapped indirect effects with normal-based and bias-corrected confidence intervals of the moderated mediation model. The confidence intervals of the interaction term Age × RSA, which represents the conditional indirect effect, show that Age moderated the mediation effect of resilience on PHQ-9, GAD-7 and PSS.

**Table 6:** Conditional Indirect effects of the moderated mediation model.

**Bootstrapped indirect effects**	**Coef**	**Confidence intervals**
IADQ → (RSA) → PHQ	0.13	0.08, 0.17 (*N*)
		0.06, 0.17 (BC)
IADQ → (RSA) → GAD	0.10	0.07, 0.14 (*N*)
		0.05, 0.13 (BC)
IADQ → (RSA) → PSS	0.10	0.06, 0.13 (*N*)
		0.08, 0.14 (BC)

*PHQ, Patient Health Questionnaire; GAD, Generalized Anxiety Questionnaire; PSS, Perceived Stress Questionnaire; RSA, Resilience Scale for Adults; IADQ, International Adjustment Disorder Questionnaire – stressful events checklist*.

## Discussion

We presented a cross-sectional study aimed to evaluate how resilience differs in an age-dependent manner, representing a key feature in older adults with respect to mental health outcomes during the COVID-19 pandemic.

Results showed that age is negatively associated with PHQ-9, GAD-7 and PSS scores. This was significant even controlling for confounding factors such as gender, region and education level. In particular, having an older age (i.e., over 60-year-old) is two-fold more negatively associated with these variables. These findings highlight that older adults report lower levels of depressive symptoms, anxiety and stress compared to a younger population. People aged 60 and over usually have a higher mortality rate and are at higher risk of developing significant complications, causing them to follow more stringent measures than the others. For these reasons an inversed trend would have been expected. Although these results might be considered as counterintuitive, a number of authors have emphasized how younger people tend to report higher levels of depressive and anxiety symptoms than older ages during pandemics (16–19). This may be explained by multiple reasons, as people aged below 60 are less likely to be retired, therefore being more preoccupied about their occupational programs and economic incomes since they might lose their job (20, 21). Furthermore, it is a matter of fact that younger people are keener on spending a consistent amount of time on social networks or other news apps (22). As a result, an information overload, also defined as “infodemic,” where fake news, racist opinions, magic potions and conspiracy theories are easily disclosed, may account for their higher scores (23). Lastly, older adults are more likely to have faced a number of major life events than their counterparts, having, therefore, a bigger wealth of experience that would allow them to face adversities more easily. In other words, older adults may better rely on their resilience when dealing with such situations. However, it is important to notice that mental health outcomes in this study were addressed using screeners that are more focused on the affective and cognitive components of anxiety and depression, rather than somatic complaints or loneliness, which are common features of psychological distress in the elderly.

According to the latest researches, COVID-19 pandemic has caused a significant increase in the prevalence of anxiety, stress and depressive symptoms (3, 4, 24). Indeed, it is not surprising that our findings showed a positive association between stressful events and depressive symptoms, anxiety and perceived stress. Notably, even controlling for potentially confounding factors like education levels, results were still significant. Indeed, according to previous studies, people with higher levels of education are more likely to develop depression and anxiety as they might be more aware of their own state of health (25).

In line with our hypothesis and consistent with previous literature, RSA scores are linearly associated with PHQ-9, GAD-7 and PSS. This means that higher levels of resilience act through a “buffering effect” on such variables, therefore mitigating COVID-19 related stressors (2).

Supporting the hypothesis that older adults have better abilities to manage calamities and to get by during difficult times, regression analyses showed that age is inversely associated with any of IADQ items and RSA scores. Results are even higher when old age is set as the independent variable, meaning that people aged 60 and over perform remarkably better at successfully overcome stressful life events such as COVID-19 pandemic. These findings are in line with previous research, as a number of authors have recognized how older people generally have high levels of resilience during difficult times, despite their own state of health, socioeconomic status and past personal experiences (26).

Our simple mediation analysis highlighted the role of COVID-19-related stressful events on resilience, reporting a positive association with RSA scores. These findings show that bigger stressful events are capable of affecting one's ability to cope with adversities. Moreover, other than having a direct effect on PHQ, GAD-7 and PSS scores, IADQ acts indirectly on these three variables. Indeed, when setting RSA as a mediating variable, regression coefficients still show a positive association with PHQ, GAD-7 and PSS scores. In other words, COVID-19-related traumatic events (e.g., losing loved ones, lockdown stringent measures, poor economic incomes) may impact directly on enhancing depressive symptoms, anxiety and perceived stress, or indirectly, lowering one's buffering effect of resilience.

The present mediating effect of RSA is different depending on the age of the subjects. In older people, resilience influences the psychopathological outcome more strongly compared to younger adults, meaning that their buffering effect is higher on depressive and anxiety symptoms and stress than their counterparts. Nevertheless, stressful events might impact differently on people aged 60 or over, with their resilience turning out to be more fragile compared to people aged under 60.

Our findings gather a strong relevance, as even if older people have stronger aptitudes to cope with difficult situations, they might experience what has been defined as a “double-burden,” having their buffering abilities compromised by the disease itself (27). Indeed, in addition to their physical vulnerability, in terms of infection risk, morbidity and mortality, they might acquire a mental vulnerability, which would then lead to alarming scenarios with worse clinical outcomes.

The present work suffers from a number of important limitations – notably related to the on-line sampling technique and due to its cross-sectional fashion. Firstly, social network-based recruitment carries a significant selection bias, as people are self-selected, without inclusion criteria, and measures are self-reported. Moreover, on-line sampling may introduce a selection bias excluding subjects with poor informatic literacy or even cognitive deficits. As a matter of fact, internet use is associated with a number of factors that promote successful aging, including cognitive functioning and higher socioeconomic status (28). Secondly, although follow-up data will be collected across time, the cross-sectional design of this study does not leave enough room for causal inferences. For these reasons, caution must be taken into account when generalizing results to the population. Thirdly, limitations concern the inability to address cognitive deficits using an on-line survey. Indeed, it is worth noticing how a number of relevant factors may negatively influence the psychological distress in older adults, including physical comorbidities, functional and cognitive impairments as well as loneliness and neglect. The counterpart of resilience is in fact frailty, defined as a condition of both physical and mental dysregulation that leads to a higher vulnerability and therefore worse health outcomes (29). However, data on physical comorbidities and dependency, that could definitely influence the selected outcomes in the elderly, were not collected.

Key strengths of our research are represented by its large sample size and its distinctive timing in collecting data, which was gathered when lockdown measures were implemented in Italy.

In conclusion, we found that older age is associated with higher levels of resilience. This would allow them to face weighty adversities such as COVID-19 pandemic more powerfully than the others. However, stressful life events may act stronger on them, compromising their buffering coping abilities, with higher rates of depression, anxiety and stress. As COVID-19 pandemic might have brought us in a new era of communication and technological progress, it will be important to develop more home-based agendas that would improve older people well-being and therefore enhance their resilience.

## Conclusion

This study addressed the age-dependent effect of resilience in mediating the impact of COVID-19 stressful events on depressive and anxiety symptoms and perceived stress. Our findings suggest that resilience plays a central role in protecting older adults from psychological distress and should therefore be taken into account in general health policies as well as treatment strategies.

## Data Availability Statement

The raw data supporting the conclusions of this article will be made available by the authors, without undue reservation.

## Ethics Statement

The studies involving human participants were reviewed and approved by University of L'Aquila. The patients/participants provided their written informed consent to participate in this study.

## Author Contributions

RR, VS, FP, and GL: conceptualization. RR: methodology, formal analysis, and data curation. RR and TJ: writing–original draft. RR, VS, TJ, FP, and GL: writing– review and editing. All authors contributed to the article and approved the submitted version.

## Conflict of Interest

The authors declare that the research was conducted in the absence of any commercial or financial relationships that could be construed as a potential conflict of interest.
